# Higher diversity of ammonia/ammonium-oxidizing prokaryotes in constructed freshwater wetland than natural coastal marine wetland

**DOI:** 10.1007/s00253-012-4430-4

**Published:** 2012-10-09

**Authors:** Yong-Feng Wang, Ji-Dong Gu

**Affiliations:** 1 Laboratory of Environmental Microbiology and Toxicology, School of Biological Sciences, The University of Hong Kong, Pokfulam Road, Hong Kong, SAR People’s Republic of China; 2 The Swire Institute of Marine Science, The University of Hong Kong, Shek O, Cape d’Aguilar, Hong Kong, SAR People’s Republic of China

**Keywords:** Anammox bacteria, Ammonia-oxidizing archaea, Ammonia-oxidizing bacteria, *amoA*, Mangrove, Diversity, Wetland, Subtropical

## Abstract

Anaerobic ammonium-oxidizing (anammox) bacteria, aerobic ammonia-oxidizing archaea (AOA), and ammonia-oxidizing bacteria (AOB) are three groups of ammonium/ammonia-oxidizing prokaryotes (AOPs) that are involved in the nitrogen cycle. This research compared the AOP communities in a constructed freshwater wetland with a natural coastal marine wetland in the subtropical Hong Kong. Both vegetated/rhizosphere and nonvegetated sediments were investigated to identify the effects of different macrophytes on the AOP communities. The polymerase chain reaction (PCR)-amplified gene fragments of 16S rRNA and archaeal and bacterial *amoA* (encoding the ammonia monooxygenase alpha subunit) were applied as molecular biomarkers to analyze the AOPs’ phylogeny and diversity. Quantitative PCR was used to determine the abundances of AOPs in the sediments. The results showed that the relatively more heterogeneous freshwater wetland contained a broader range of phylotypes, higher diversity, more complex community structures, and more unevenly distributed abundances of AOPs than the coastal wetland. The effects of vegetation on the community structures of AOPs were plant-specific. The exotic *Typha angustifolia* affected the community structures of all AOPs and enhanced their abundances in the rhizosphere region. Both *Phragmites australis* and *Cyperus malaccensis* showed some effects on the community structures of AOB, but minimal effects on those of anammox bacteria or AOA. *Kandelia obovata* had almost no detectable effect on all AOPs due to their smaller size. This study suggested that the freshwater and coastal marine wetlands may have different contributions to the inorganic N removal due to the variations in AOP communities and plant types.

**Electronic supplementary material:**

The online version of this article (doi:10.1007/s00253-012-4430-4) contains supplementary material, which is available to authorized users.

## Introduction

Ammonium/ammonia-oxidizing prokaryotes (AOPs) contribute to ammonia oxidation in the global nitrogen cycle, including anaerobic ammonium oxidation (anammox) and aerobic ammonia oxidization. Anammox, the process in which ammonium is transformed with nitrite to dinitrogen gas (N_2_), was first discovered from a wastewater treatment plant (Mulder et al. 1995). All known anammox bacteria belong to five genera in the phylum *Planctomycetales*: *Candidatus* Brocadia (Strous et al. 1999), *Ca.* Kuenenia (Schmid et al. 2000), *Ca.* Scalindua (Schmid et al. 2003), *Ca.* Anammoxoglobus (Kartal et al. 2007), and *Ca.* Jettenia (Quan et al. 2008). No pure cultures of anammox bacteria have been obtained due to their notoriously slow growth rate (Jetten et al. 1998, 2009). In addition to the application to inorganic N removal in sewage treatment (Kartal et al. 2010), anammox bacteria have been demonstrated to play a significant role in marine environments, including the anoxic basin of the Black Sea (Kuypers et al. 2003), the Benguela and Peru upwelling systems (Kuypers et al. 2005; Lam et al. 2009), the sediments of the South China Sea and Jiaozhou Bay of China (Dang et al. 2010; Hong et al. 2011), coastal and estuary sediments (Dale et al. 2009; Li et al. 2011b), oil fields (Li et al. 2010a), and even polar marine sediments and sea ice (Rysgaard and Glud 2004). It was estimated that anammox bacteria might be responsible for more than 50 % of global nitrogen losses from the oceans (Brandes et al. 2007). In addition to marine environments, anammox bacteria were also detected in freshwater and terrestrial environments such as lakes (Hamersley et al. 2009; Schubert et al. 2006), rivers (Zhang et al. 2007), terrestrial soils (Penton et al. 2006), paddy fields (Wang and Gu 2012), groundwater (Clark et al. 2008), hot springs (Jaeschke et al. 2009), and coastal mangrove wetlands (Cao et al. 2011a; Li et al. 2011a, b). Functional genes encoding hydrazine oxidoreductase (*hzo*), nitrite reductase (*nir*), and hydrazine synthase (*hzs*) have been used to detect anammox bacteria in natural environments (Harhangi et al. 2012; Li et al. 2010b; Schmid et al. 2008), but the more widely used molecular biomarker is still the 16S rRNA gene (Li and Gu 2011).

Aerobic ammonia oxidation is the first and rate-limiting step in nitrification (Purkhold et al. 2000), through which ammonia is oxidized with oxygen to nitrite by two phylogenetically distinct microbial groups: ammonia-oxidizing bacteria (AOB) and ammonia-oxidizing archaea (AOA). AOB were found capable of catalyzing this process more than one century ago (Winogradsky 1890). To date, all known AOB fall into two phylogenetic lineages within the β- and γ-Proteobacteria (reviewed by Kowalchuk and Stephen 2001). In addition to AOB, the newly discovered AOA are also able to oxidize ammonia to nitrite under aerobic conditions (Könneke et al. 2005). AOA was initially classified into the phylum *Crenarchaeota* (Könneke et al. 2005) and later into the newly proposed phylum *Thaumarchaeota* based on genomic level comparison (Brochier-Armanet et al. 2008; Pester et al. 2011). Although AOB and AOA belong to different domains, both of them contain homologous ammonia monooxygenase (Amo), which oxidizes ammonia with oxygen to hydroxylamine. The gene *amoA*, encoding the alpha subunit of Amo, is widely used as a functional marker to analyze the phylogeny and abundance of AOB and AOA in the environments (Francis et al. 2005; Rotthauwe et al. 1997).

Wetlands are an important ecosystem for their ecological functions by offering habitats (Nagelkerken et al. 2008), supporting abundant life (Nagelkerken et al. 2008), possessing high diversity (Gopal and Ghosh 2008), and purifying water (Michael 2010). Plants are the most important primary producers in wetlands. In addition to being highly productive, plants affect nutrient cycling in the wetland by transferring oxygen to the vicinity of roots and form a special microenvironment around the rhizosphere that is different from the bulk soil and the nonvegetated soil (Zhang et al. 2009). A number of studies have been carried out on AOP communities in wetlands, particularly in the rhizospheres (Cao et al. 2011a, b, 2012; Dale et al. 2009; Herrmann et al. 2008, 2009; Li et al. 2011b). However, the related research reports are still very limited. For example, are AOP communities in the coastal wetland and the freshwater wetland different? Is the effect of the rhizosphere on the AOP community plant-specific? The coastal and the freshwater wetlands are essentially different because they are respectively under the influence of seawater and freshwater of different physiochemical characteristics. Because AOPs have been shown to have separate niches (Schleper 2010), it can be assumed that AOP communities in different types of wetlands are different. In addition, AOP communities in vegetated sediments might be different from the nonvegetated ones. In order to verify our hypothesis, we investigated AOP communities in the rhizosphere and the nonvegetated sediments in a natural coastal wetland and a constructed freshwater wetland in the subtropical Hong Kong using the molecular biomarkers 16S rRNA gene and archaeal and bacterial *amoA* genes. Through this study, we would like to further our understanding and knowledge about the effects of the wetland type and the rhizospheres of different plants on AOP communities.

## Materials and methods

### Description of sites and sampling

The natural coastal wetland is located at Tai O (22°14′59″ N, 113°51′43″ E) in subtropical Hong Kong (Supplement material Fig. S1), restored from deserted salt pans about 40 years ago with mangrove trees *Kandelia obovata* being the dominant plants. Samples were taken by layers at the depths of 0–2, 4–6, 9–11, 14–16, 19–21, 24–26, 29–31, and 39–41 cm at both vegetated and nonvegetated sites on March 13, 2009, the end of dry season and the beginning of wet season in Hong Kong. The nonvegetated sampling site was about 10 m away from the vegetated one. The overlying water at each site was also collected for physiochemical analyses in laboratory.

The constructed freshwater wetland is located at Yuen Long (22°27′20″ N, 114°2′43″ E) in Hong Kong (Supplement material Fig. S1), transformed from three fishponds in 2003 for creating a wetland habitat for wild birds, amphibians, and dragonflies. Aquatic macrophytes *Phragmites australis*, *Typha angustifolia*, and *Cyperus malaccensis* were dominant plants at the upstream, middle stream, and downstream of the wetland, respectively. The input water of the wetland was made up of runoff from adjacent hills mixed with a small fraction of wastewater from nearby villages. Sediment samples were respectively taken from the sites grown with *P. australis*, *T. angustifolia*, and *C. malaccensis* on March 24, 2009. At each site, the rhizosphere sediments of the plants and the adjacent nonvegetated sediments about 10 m away were collected. The overlying water at each site was also collected for physiochemical analyses.

All sediment and water samples were immediately put into ice boxes and transported back to the laboratory shortly after collection. In the laboratory, samples for subsequent molecular studies were kept at −80 °C and samples for physiochemical analysis were processed immediately.

### Physiochemical analyses

The temperature, pH, and redox potentials of the sediments and water were measured in situ with the IQ160 pH meter (with ORP electrode) (IQ Scientific Instruments, Inc.). The conductivity, turbidity, and salinity of the water were measured in situ with the Water Quality Checker U-10 (HORIBA). Inorganic N of the sediments was extracted with 2 M KCl in 1:4 ratio (sediment to solution) for 1 h. Ammonia-N, nitrite-N, and nitrate-N of the extracts, as well as of the overlying water collected from the two wetlands, were determined with the Lachat QuikChem 8000 Flow Injection Analyzer (Lachat Instruments Inc.). The measuring procedures were in accordance with the manual of the instrument. Sediment dry weights were measured after drying in an oven at 105 °C for 24 h till constant weight was achieved.

### Sediment DNA extraction and PCR amplification

Sediment total DNA was extracted using the SoilMaster DNA Extraction Kit (Epicentre Biotechnologies, Madison, WI, USA) according to the manual of the manufacturer within 1 month after sample collection. DNA was finally eluted with 250 μl of TE buffer included in the kit. The extracted sediment DNA was then used for subsequent molecular analysis and stored at −20 °C after use. Anammox bacterial 16S rRNA gene fragments and archaeal and bacterial *amoA* genes were amplified using the GoTaq Flexi DNA Polymerase Kit (Promega, Hong Kong) with different protocols described below.

The 16S rRNA gene fragments of anammox bacteria were amplified using Amx368F (5′-TTCGCAATGCCCGAAAGG-3′) and Amx820R (5′-AAAACCCCTCTACTTAGTGCCC-3′). The optimized polymerase chain reaction (PCR) mixture contained, in a final volume of 50 μl, as follows: 1.5 μl of DNA (20 ng μl^−1^), 10 μl of 5× GoTaq Flexi Buffer (Promega, Hong Kong), 4 μl of MgCl_2_ (25 mM; Promega, Hong Kong), 1 μl of deoxyribonucleotide triphosphates (dNTPs; 10 mM of each; Promega, Hong Kong), 1 μl of each forward and reverse primers (20 μM), 0.25 μl of GoTaq Flexi Polymerase (5 U μl^−1^; Promega, Hong Kong), and 5 μl of bovine serum albumin (BSA; 0.1 %). PCR conditions were set as follows: 94 °C for 4 min; 30 cycles of 95 °C for 45 s, 59 °C for 50 s, followed by 72 °C for 1 min; and finally, 72 °C for 15 min.

The archaeal *amoA* genes were amplified using the primers Arch-*amoA*F (5′-STAATGGTCTGGCTTAGACG-3′) and Arch-*amoA*R (5′-GCGGCCATCCATCTGTATGT-3′). Based on the standard procedures in the manufacturer’s instructions and results of previous studies (Francis et al. 2005), the optimized PCR mixture contained, in a final volume of 50 μl, as follows: 1.5 μl of DNA (20 ng μl^−1^), 10 μl of 5× GoTaq Flexi Buffer (Promega, Hong Kong), 3 μl of MgCl_2_ (25 mM; Promega, Hong Kong), 1 μl of dNTPs (10 mM of each; Promega, Hong Kong), 1 μl of each forward and reverse primers (20 μM), 0.25 μl of GoTaq Flexi Polymerase (5 U μl^−1^; Promega, Hong Kong), and 5 μl of BSA (0.1 %). PCR conditions were set as follows: 95 °C for 5 min; 30 cycles of 94 °C for 45 s, 53 °C for 1 min, and 72 °C for 1 min; and finally, 72 °C for 15 min.

The bacterial *amoA* genes were amplified using the primers *amoA*-1F (5′-GGGGGTTTCTACTGGTGGT-3′) and *amoA*-2R (5′-CCCCTCKGSAAAGCCTTCTTC-3′). The optimized PCR mixture contained, in a final volume of 50 μl, as follows: 1.5 μl of DNA (20 ng μl^−1^), 10 μl of 5× GoTaq Flexi Buffer (Promega, Hong Kong), 2.5 μl of MgCl_2_ (25 mM; Promega, Hong Kong), 1 μl of dNTPs (10 mM of each; Promega, Hong Kong), 1 μl of each forward and reverse primers (20 μM), 0.25 μl of GoTaq Flexi Polymerase (5 U μl^−1^; Promega, Hong Kong), and 5 μl of BSA (0.1 %). PCR conditions were set as follows: 94 °C for 3 min; 30 cycles of 94 °C for 45 s, 55 °C for 45 s, and 72 °C for 50 s; and finally, 72 °C for 10 min.

PCR products were checked by electrophoresis on 1 % agarose gel staining with ethidium bromide (0.5 μg ml^−1^).

### Cloning and sequencing

All the PCR-amplified products were purified through cutting gel bands with a Gel Advanced™ Gel Extraction System (Viogene-Bio Tek Co., Taiwan, Republic of China) according to the manufacturer’s instructions. The purified products were ligated into the pMD18 T-vector (Takara, Japan) and then transformed into the host *Escherichia coli* DH5α competent cell (Takara, Japan) in accordance with the manufacturer’s instructions. Clones were randomly selected and insertions of an appropriate-sized DNA fragment were determined by PCR amplification with the primer set M13F and M13R. Positive clones were then sequenced with ABI 3730xl DNA Analyzer (Applied Biosystems) at the Genome Research Centre of The University of Hong Kong.

### Analyses of phylogeny, rarefaction, richness, and diversity

Sequences were analyzed against those in the GenBank with BLAST and Ribosomal Database Project II (Altschul et al. 1990; Cole et al. 2005). They were aligned and phylogenetic trees were constructed using MEGA, version 5.1 (Tamura et al. 2011). For anammox bacteria, clones with more than 99 % nucleotide sequence similarity were grouped into the same operational taxonomic unit (OTU), and their representative sequences were used for phylogenetic analysis. For AOA and AOB, clones with more than 97 % putative amino acid sequence similarity were grouped into the same OTU, and their representative sequences were used for phylogenetic analysis. Phylogenetic trees were constructed with the neighbor-joining method with 1,000 bootstrap repetitions to estimate the confidence of the tree topologies.

To compare anammox bacterial 16S rRNA and archaeal and bacterial *amoA* gene-based richness and diversity within each clone library, rarefaction, Chao nonparametric richness, and Shannon indices of diversity were calculated using DOTUR (Schloss and Handelsman 2005).

### Real-time quantitative PCR analysis

The abundances of anammox bacterial 16S rRNA genes and archaeal and bacterial *amoA* genes were determined in triplicate with real-time quantitative PCR amplification using a FastStart Universal SYBR Green Master (Rox) Kit (Roche, Germany). Real-time quantitative PCR was performed in 96-well optical plates placed in the ABI PRISM® 7000 Sequence Detection System (Applied Biosystems). The primer set composed of Amx368F and Amx820R was used for the amplification of 16S rRNA genes of anammox bacteria. The primer set composed of Arch-*amoA*F and Arch-*amoA*R was used for the amplification of the *amoA* genes of AOA, and the primer set composed of *amoA*-1F and *amoA*-2R was used for the amplification of the *amoA* genes of AOB. The final reaction volume was 20 μl and the reaction composition and cycling conditions were in accordance with the manual.

The specificity of the PCR amplification was determined by the melting curve and gel electrophoresis. Cycle thresholds were determined by comparing with the standard curves constructed using a 10-fold serial dilution (10^2^–10^7^ gene copies μl^−1^) of the newly extracted plasmids containing the corresponding gene fragments. Relative copy numbers among target groups were evaluated, and some replicates of apparent discrepancy were excluded in order to decrease standard error. The correlation coefficient *R* ^2^ values were >0.97 for all of the standard curves.

### PCoA analysis

Fast UniFrac provides a suite of tools for the comparison of microbial communities using phylogenetic information (Hamady et al. 2009). To compare microbial communities in different environments, the phylogenetic trees of anammox bacteria, AOA, and AOB were analyzed online using Jackknife environment clusters analysis (UPGMA algorithm with 100 replicates Jackknife supporting test) and principal coordinates analysis (PCoA) on the website of Fast UniFrac (http://bmf2.colorado.edu/fastunifrac/).

### Nucleotide sequence accession numbers

The anammox bacterial 16S rRNA gene sequences determined in this study are available in GenBank under accession numbers JQ886186 to JQ886236, AOA *amoA* gene sequences under accession numbers JQ886237 to JQ886313, and AOB *amoA* gene sequences under accession numbers JQ886314 to JQ886397.

## Results

### Physiochemical characteristics of sediment samples

When considering wetland type, the coastal marine wetland and the freshwater wetland had very different physiochemical characteristics (Table 1). Firstly, the average salinity, conductivity, and turbidity of the overlying water from the coastal wetland were 2.97 ‰, 45.40 mS cm^−1^, and 5.9 NTU, respectively, much higher than those from the freshwater wetland (0.05 ‰, 1.23 mS cm^−1^, and 1.7 NTU, respectively). Secondly, the average NH_4_^+^ concentration of the sediments from the coastal wetland was 1.68 μg N g^−1^ DW, higher than 0.36 μg N g^−1^ DW of those from the freshwater wetland. However, the average NO_2_^−^ and NO_3_^−^ concentrations of the sediments from the coastal wetland were 0.02 and 0.12 μg N g^−1^ DW, respectively, lower than those from the freshwater wetland (0.15 and 0.18 μg N g^−1^ DW, respectively). Thirdly, the average pH of the sediments from the coastal wetland was 7.1, much higher than 5.4 of those from the freshwater wetland.

**Table 1:** The physiochemical characteristics of sediments and overlying water at the two wetlands

	Sample ID	Sample source	Ammonium (μg N g^−1^ dry weight)	Nitrite (μg N g^−1^ dry weight)	Nitrate (μg N g^−1^ dry weight)	Temperature (°C)	pH	Redox Potential (mV)	Salinity (‰)	Conductivity (mS cm^−1^)	Turbidity (NTU)
Freshwater wetland	PAUw	Overlying water^a^	1.766	0.006	0.579	22.6	7.3	224.0	0.06	1.47	2.0
	PAUn	Nonvegetated	0.016	0.092	0.056	22.2	6.3	−19.5			
	PAUr	Rhizosphere	0.248	0.167	0.258	21.8	4.6	290.0			
	TANw	Overlying water^a^	0.058	0.003	0.037	23.8	6.7	207.0	0.03	0.75	3.0
	TANn	Nonvegetated	1.650	0.298	0.402	22.5	5.9	51.7			
	TANr	Rhizosphere	0.004	0.086	0.096	21.8	5.7	234.0			
	CMAw	Overlying water^a^	0.884	0.012	0.027	24	4.3	278.0	0.06	1.48	0.0
	CMAn	Nonvegetated	0.258	0.124	0.121	21.8	5.6	289.0			
	CMAr	Rhizosphere	UD	0.154	0.145	22.1	4.1	284.0			
Coastal wetland	KOBnw	Overlying water^a^	0.159	0.002	0.074	20.1	8.0	200.0	3.12	47.40	3.0
	KOBn00	0–2 cm layer	2.186	UD	0.105	18.7	7.2	155.0			
	KOBn05	4–6 cm layer	1.116	0.012	0.135	20.3	7.2	118.0			
	KOBn10	9–11 cm layer	0.554	0.014	0.091	20.8	7.2	−84.0			
	KOBn15	14–16 cm layer	1.283	UD	0.098	19.5	7.4	−84.0			
	KOBn20	19–21 cm layer	2.275	0.007	0.083	19.6	7.5	−92.0			
	KOBn25	24–26 cm layer	2.865	0.025	0.114	19.6	7.5	−108.0			
	KOBn30	29–31 cm layer	3.615	0.015	0.085	19.9	7.5	−90.0			
	KOBn40	39–41 cm layer	7.021	0.012	0.094	20.0	7.4	−117.9			
	KOBrw	Overlying water^a^	0.087	UD	0.018	21.2	7.7	195.0	2.82	43.40	8.8
	KOBr00	0–2 cm layer	1.884	0.053	0.239	23.4	6.9	−28.4			
	KOBr05	4–6 cm layer	0.595	0.018	0.130	22.1	6.7	−66.0			
	KOBr10	9–11 cm layer	0.531	0.016	0.136	21.2	6.8	−70.4			
	KOBr15	14–16 cm layer	0.597	0.013	0.145	21.0	6.8	−71.7			
	KOBr20	19–21 cm layer	0.578	0.007	0.107	21.0	6.9	−78.2			
	KOBr25	24–26 cm layer	0.676	0.019	0.092	21.1	6.8	−93.4			
	KOBr30	29–31 cm layer	0.483	0.022	0.122	21.0	6.8	−156.2			
	KOBr40	39–41 cm layer	0.544	0.016	0.155	21.0	6.9	−147.9			
Mean ± SD	Nonvegetated sediments		2.08 ± 1.98	0.05 ± 0.09	0.13 ± 0.09	20.5 ± 1.2	7.0 ± 0.7	1.7 ± 134.4			
	Vegetated sediments		0.56 ± 0.50	0.05 ± 0.06	0.15 ± 0.05	21.6 ± 0.8	6.3 ± 1.0	8.7 ± 171.8			
	Freshwater wetland		0.36 ± 0.64	0.15 ± 0.08	0.18 ± 0.13	22.1 ± 0.3	5.4 ± 0.8	188.2 ± 136.8	0.05 ± 0.02	1.23 ± 0.42	1.7 ± 1.5
	Coastal wetland		1.68 ± 1.73	0.02 ± 0.01	0.12 ± 0.04	20.6 ± 1.1	7.1 ± 0.3	−63.4 ± 84.1	2.97 ± 0.21	45.40 ± 2.83	5.9 ± 4.1

The last two numbers of the sample ID indicate the depth of the sample

*PAU* site with *P. australis*, *TAN* site with *T. angustifolia*, *CMA* site with *C. malaccensis*, *w* overlying water, *r* rhizosphere, *n* nonvegetated sediment, *UD* under detection

^a^The unit of ammonium, nitrite, and nitrate of overlying water is milligrams of N per liter

When taking into account vegetation, vegetated sediments (including the rhizosphere in this study) had apparently different physiochemical properties from those of nonvegetated ones (Table 1). Firstly, the average NH_4_^+^ concentration of the vegetated sediments was 0.56 μg N g^−1^ DW, which was lower than 2.08 μg N g^−1^ DW of nonvegetated ones. Secondly, the average pH of the vegetated sediments was 6.3, which was lower than 7.0 of the nonvegetated ones. Thirdly, in the freshwater wetland, redox potentials of the vegetated sediments were much higher than those of the corresponding nonvegetated ones (Table 1). In the coastal wetland, however, redox potentials did not show obvious differences between the vegetated and nonvegetated sediments (Table 1).

### Phylogeny of anammox bacteria, AOA, and AOB

**Fig. 1:**
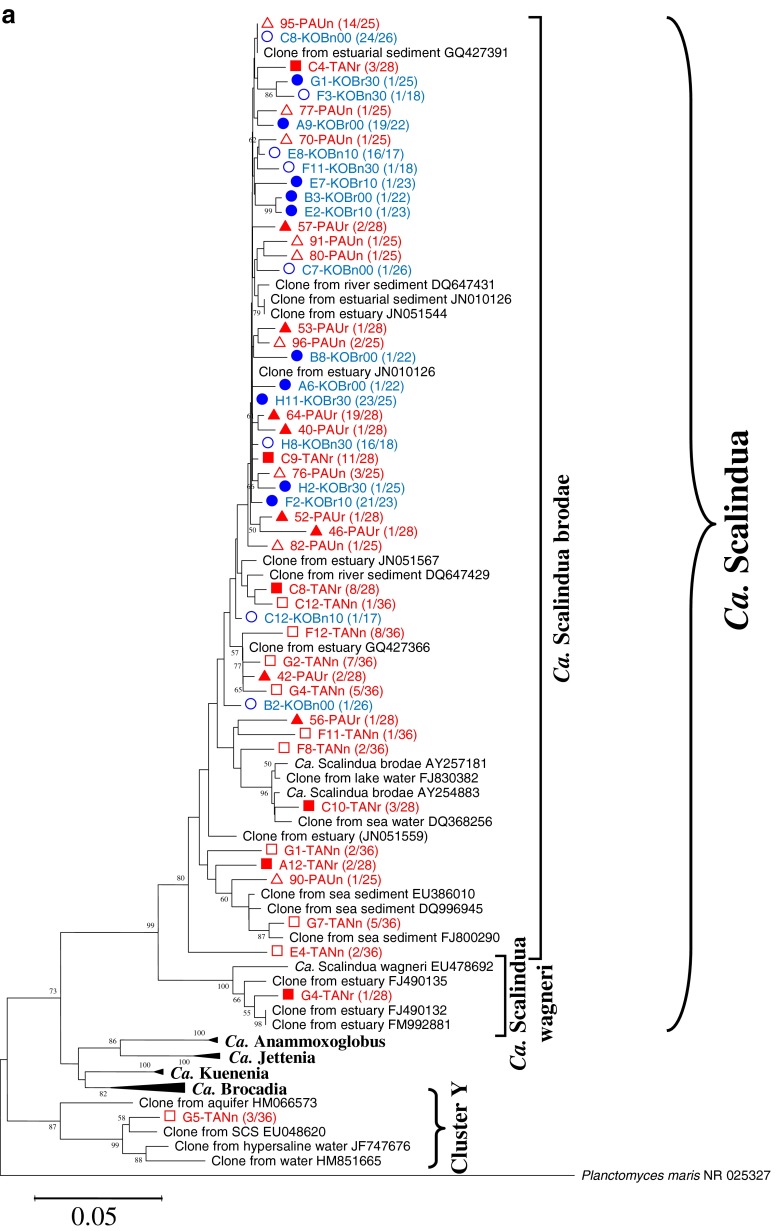

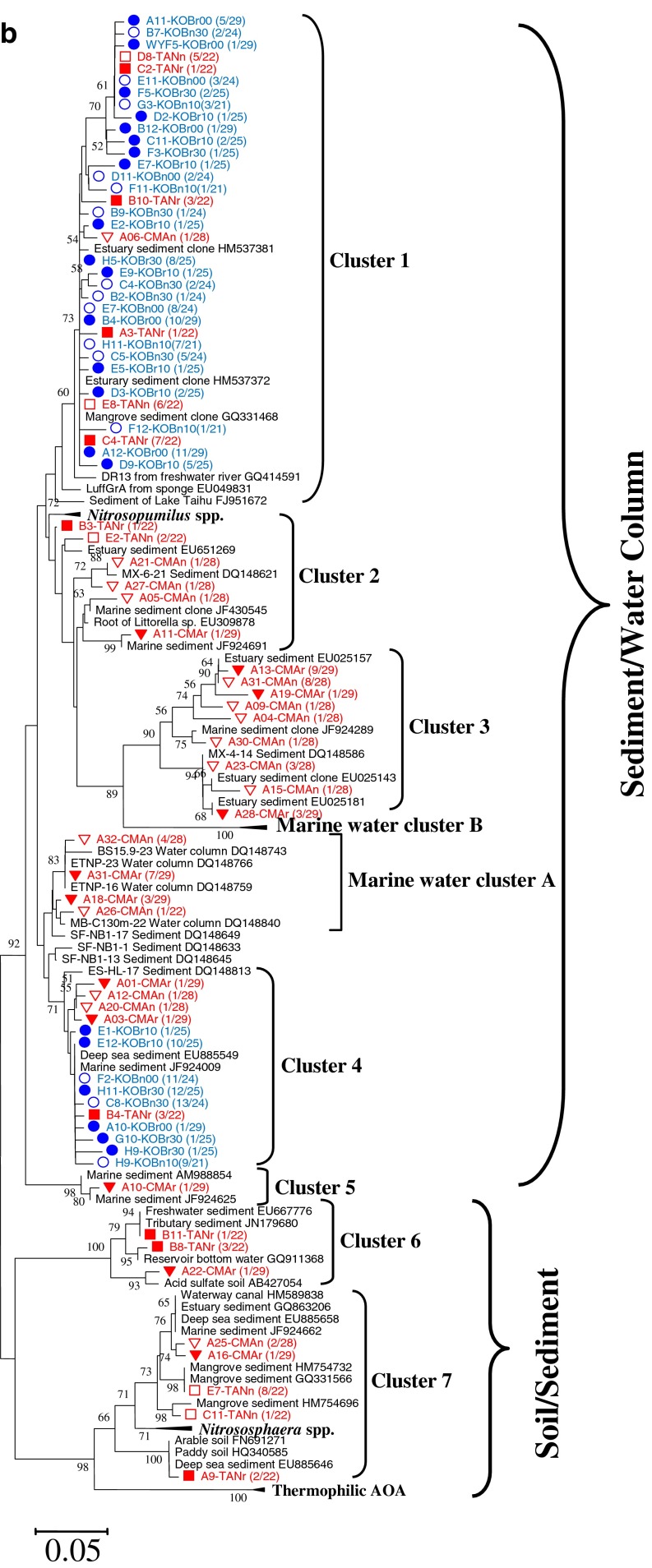

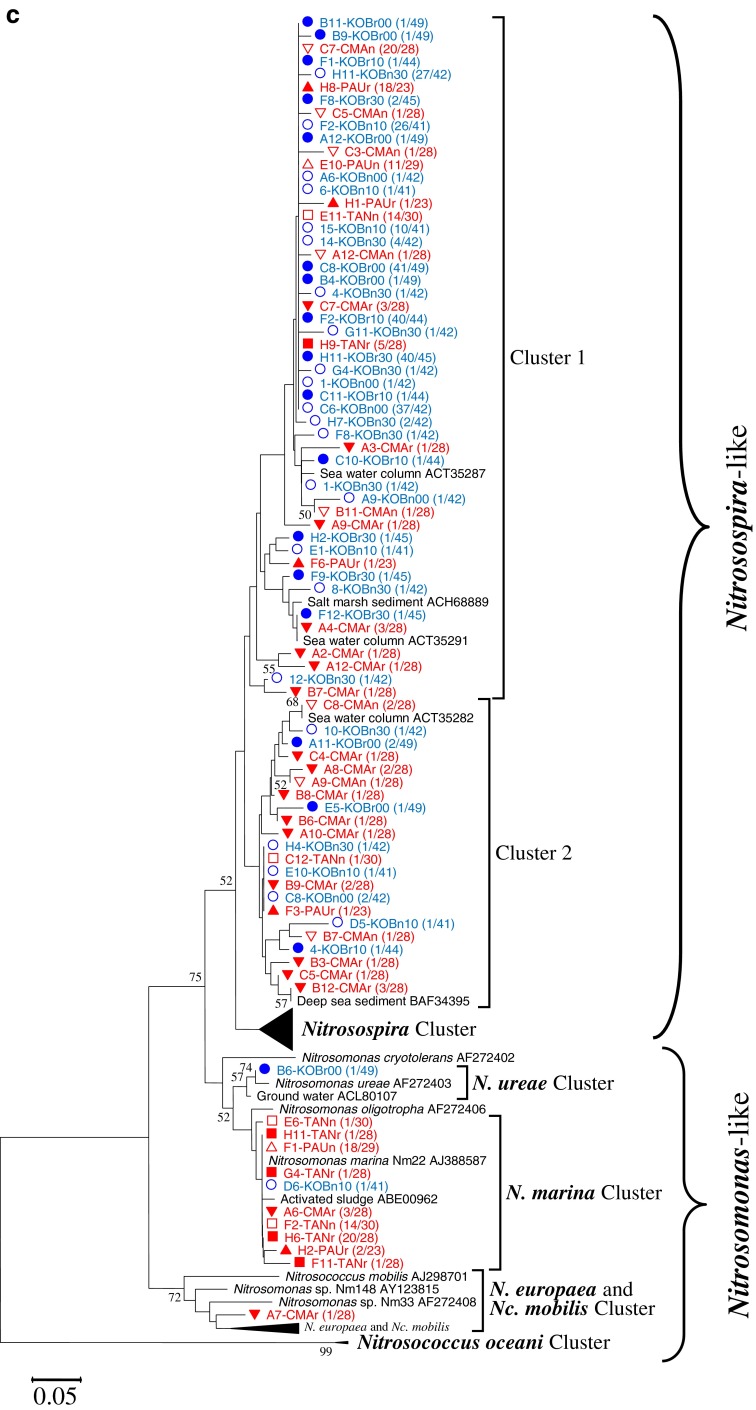
Phylogenetic trees based on nucleotide sequences of 16S rRNA gene-amplified fragments of anammox bacteria (**a**) and the *amoA* gene sequences of AOA (**b**) and AOB (**c**). The tree for anammox bacteria was reconstructed based on partial 16S rRNA sequences (477 nucleotides). The trees for AOA and AOB were reconstructed based on partial AmoA sequences (198 and 150 amino acids for AOA and AOB, respectively). For anammox bacteria, clones with more than 99 % nucleotide sequence similarity were grouped into the same OTU; for AOA and AOB, clones with more than 97 % putative amino acid sequence similarity were grouped into the same OTU, and their representative sequences were used for phylogenetic analysis. The numbers of OTUs and total sequences in the libraries are shown in *parentheses*. Phylogenetic trees were constructed with the neighbor-joining method with 1,000 bootstrap repetitions to estimate the confidence of the tree topologies. Bootstrap values (>50 %) are indicated at the branch points. The *solid and hollow circles with blue color* represent the vegetated and nonvegetated sediments of *K. obovata*, respectively. The *solid and hollow triangles with red color* represent the rhizospheres and nonvegetated sediments of *P. australis*, respectively. *The solid and hollow squares with red color* represent the rhizospheres and nonvegetated sediments of *T. angustifolia*, respectively. The *solid and hollow inverted triangles with red color* represent the rhizospheres and nonvegetated sediments of *C. malaccensis*, respectively

The phylogenetic tree of anammox bacteria in the two wetlands amplified by using 16S rRNA genes is shown in Fig. 1a. A total of 248 clones were acquired from all the sediment samples of the two wetlands. Clones with more than 99 % nucleotide sequence similarity were grouped into the same OTU, and their representative sequences were used for phylogenetic analysis. The phylogenetic tree shows that both wetlands, especially the coastal one, had a narrow range of anammox bacteria phylotypes. All the clones of the coastal wetland (131) were affiliated with *Ca.* Scalindua brodae. Although the freshwater wetland showed a relatively broader range of phylotypes, the majority of the clones (113 out of 117) fell into *Ca.* Scalindua brodae, only one clone affiliated with *Ca.* Scalindua wagneri, and three clones with a unique new cluster Y.

The phylogenetic tree of AOA in the two wetlands analyzed using putative amino acid sequences from the amplified *amoA* genes is shown in Fig. 1b. A total of 249 archaeal *amoA* clones were acquired from all the sediment samples of the two wetlands. Clones with more than 97 % putative amino acid sequence similarity were grouped into the same OTU, and their representative sequences were used for phylogenetic analysis. This study adopted the classifying method of Francis et al. (2005), in which AOA sequences were classified by habitats and three clades were defined: sediments, water column, and soil/sediment. The coastal wetland had a narrower range of phylotypes than the freshwater wetland. All clones (148) recovered from the coastal wetland fell into clusters 1 and 4 within the sediments clade. The freshwater wetland showed much broader AOA phylotypes. The sequences retrieved from the freshwater wetland fell into sediment clade (70 out of 101), soil/sediment clade (19 out of 101), and water column clade (12 out of 101). In more detail, they respectively belonged to clusters 1 to 7, and the water column belonged to cluster A (Fig. 1b).

The phylogenetic tree of AOB in the two wetlands analyzed using putative amino acid sequences of the amplified *amoA* genes is shown in Fig. 1c. A total of 429 bacterial *amoA* gene clone sequences were acquired from all the samples of the two wetlands. Clones with more than 97 % putative amino acid sequence similarity were grouped into the same OTU, and their representative sequences were used for phylogenetic analysis. The majority of the clones (365 out of 429) fell into the *Nitrosospira*-like genus, and the rest (64 out of 429) fell into the *Nitrosomonas*-like genus. In the *Nitrosomonas*-like genus, all clones fell into known clusters in which pure cultures have been obtained. In the *Nitrosospira*-like genus, all the retrieved sequences were affiliated with two unknown clusters, provisionally named cluster 1 (336 clones) and cluster 2 (29 clones). As shown in the AOB phylogenetic tree, the freshwater wetland had a broader range of phylotypes than the coastal marine wetland. For the coastal marine wetland, almost all sequences (261 out of 263) concentrated within the *Nitrosospira*-like genus, and only two sequences belonged to the *Nitrosomonas*-like genus. However, for the freshwater wetland, although the majority of them (104 out of 166) were affiliated with the *Nitrosospira*-like genus, more than one third of the sequences (62 out of 166) fell into the *Nitrosomonas*-like genus.

### Diversity of anammox bacteria, AOA, and AOB

The Shannon index of anammox bacteria in the freshwater wetland was 1.60 ± 0.36, much higher than 0.37 ± 0.11 in the coastal wetland (Table 2). This result is in agreement with the anammox bacterial phylogenetic tree (Fig. 1a), showing that the freshwater wetland contained a broader range of phylotypes. The rarefaction curve also shows that the freshwater wetland had more OTUs than the coastal wetland (Supplement material Fig. S2).

**Table 2:** Observed and estimated richness of anammox bacterial 16S rRNA and archaeal and bacterial *amoA* gene libraries

	Sample ID	Anammox bacteria				AOA				AOB			
		No. of clones sequenced	No. of OTUs (1 %)	Chao	Shannon *H*′	No. of clones sequenced	No. of OTUs (3 %)	Chao	Shannon *H*′	No. of clones sequenced	No. of OTUs (3 %)	Chao	Shannon *H*′
Freshwater wetland	PAUn	25	9	16.5	1.55					29	2	2	0.66
	PAUr	28	8	11.3	1.24					23	5	6.5	0.81
	TANn	36	10	10.3	2.09	22	5	5	1.42	30	4	5	0.94
	TANr	28	6	6	1.51	22	9	12	1.96	28	5	8	0.90
	CMAn					28	15	42.5	2.37	28	8	15.5	1.14
	CMAr					29	11	32	1.99	28	18	40	2.76
	Mean ± SD				1.60 ± 0.36				1.93 ± 0.39				1.20 ± 0.78
Coastal wetland	KOBn00	26	3	4.0	0.32	24	4	4.0	1.19	42	5	6.5	0.52
	KOBn10	17	2	2.0	0.22	21	5	6.0	1.30	41	7	17	1.09
	KOBn30	18	3	4.0	0.43	24	6	6.3	1.34	42	12	30	1.45
	KOBr00	22	4	7.0	0.55	29	6	9.0	1.39	49	8	15.5	0.76
	KOBr10	23	3	4.0	0.36	25	10	15.0	1.87	44	5	11	0.43
	KOBr30	25	3	4.0	0.33	25	6	7.5	1.31	45	5	6.5	0.5
	Mean ± SD				0.37 ± 0.11				1.40 ± 0.24				0.79 ± 0.40

OTUs were defined as 1 % difference in nucleotide sequence alignment for anammox bacteria and 3 % difference in deduced amino acid sequence alignment for AOA and AOB, determined using DOTUR; Chao-estimated richness and Shannon index were also calculated using DOTUR. The last two numbers of the sample ID indicate the depth of the sample

*PAU* site growing *P. australis*, *TAN* site growing *T. angustifolia*, *CMA* site growing *C. malaccensis*, *r* rhizosphere for freshwater wetland or vegetated sediment for coastal wetland, *n* nonvegetated sediment

The Shannon index of AOA in the freshwater wetland was 1.93 **±** 0.39, higher than 1.40 ± 0.24 in the coastal wetland (Table 2). This result is in agreement with the AOA phylogenetic tree that the freshwater wetland had a broader range of AOA phylotypes (Fig. 1b). The rarefaction curve also shows that the freshwater wetland had more OTUs than the coastal wetland (Supplement material Fig. S2).

The Shannon index of AOB in the freshwater wetland was 1.20 **±** 0.78, higher than 0.79 **±** 0.40 in the coastal wetland (Table 2). This result is in agreement with the AOB phylogenetic tree in which the freshwater wetland had broader phylotypes than the coastal wetland (Fig. 1c). The rarefaction curve also shows that the freshwater wetland had more AOB OTUs than the coastal wetland (Supplement material Fig. S2).

### Community structure comparisons of anammox bacteria, AOA, and AOB

**Fig. 2:**
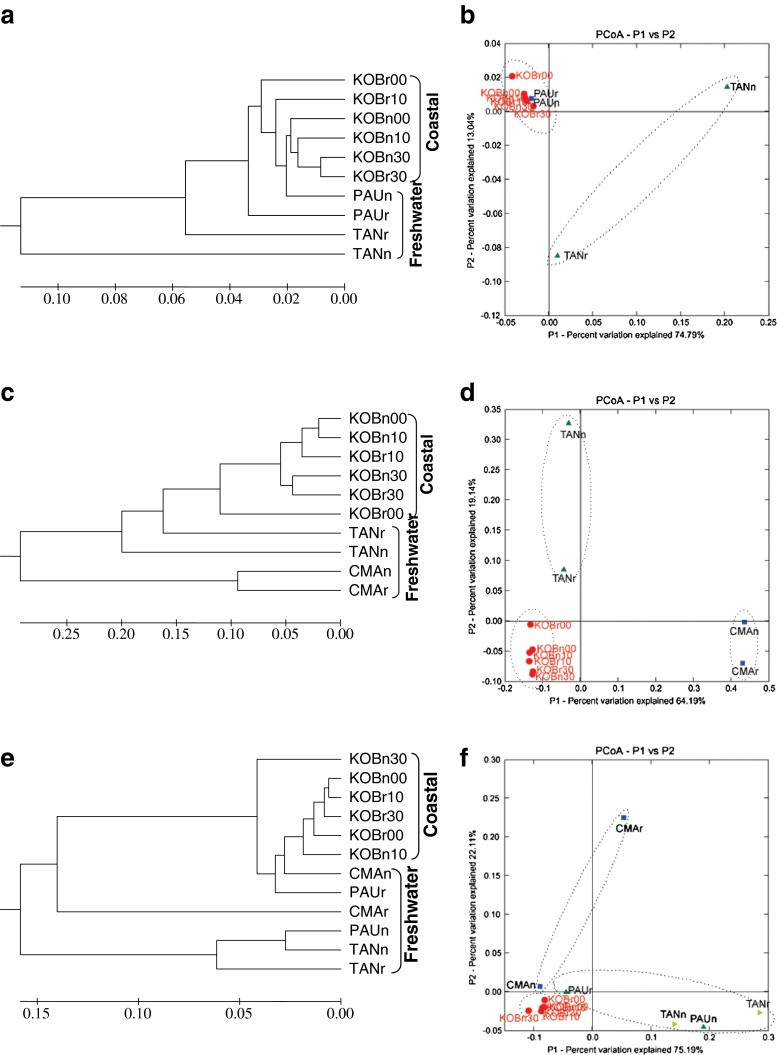
Jackknife environment clusters analysis (UPGMA algorithm with 100 replicates Jackknife supporting test) and PCoA using the online software Fast UniFrac based on the distance metric of 16S rRNA genes of anammox bacteria (**a**, **b**), deduced archaeal AmoA amino acid sequences (**c**, **d**), and deduced bacterial AmoA amino acid sequences (**e**, **f**), based on the sequence abundance data. For the sample ID, *PAU* indicates the site with *P. australis*, *TAN* indicates the site with *T. angustifolia*, *CMA* indicates the site with *C. malaccensis*, *KOB* indicates the site with *K. obovata*, *r* indicates rhizosphere or vegetated sediment, *n* indicates nonvegetated sediment, and the last two numbers indicate the depth of the sample

AOP community structure comparisons between the coastal and freshwater wetlands were also analyzed with Jackknife environment clusters analysis and PCoA (Fig. 2). For all AOPs, samples from the coastal wetlands were clustered together, while samples from the freshwater wetland scattered and were not clustered with the samples from the coastal wetland, suggesting that AOP community structures in the two wetlands were different, i.e., the wetland type had an obvious effect on the community structures of AOPs.

In the coastal wetland, the vegetated and nonvegetated samples were clustered together, indicating that community structures of AOPs in the vegetated sediments of *K. obovata* and nonvegetated ones were similar. In the freshwater wetland, the situations were much more complex. For anammox bacteria (Fig. 2a, b), the rhizosphere and nonvegetated samples of *P. australis* site were clustered together, suggesting that the community structures of anammox bacteria in the two samples were similar. However, the rhizosphere and nonvegetated samples of *T. angustifolia* site were not clustered closely, indicating that the community structures of anammox bacteria in the two samples were different. For AOA (Fig. 2c, d), the rhizosphere and nonvegetated sediment samples of the *C. malaccensis* site were not clustered closely, indicating that the community structures of the vegetated and nonvegetated sediments were somewhat different. The rhizosphere and nonvegetated sediments of the *T. angustifolia* site were not clustered together at all, suggesting that the AOA community structures of the sediments were quite different. For AOB (Fig. 2e, f), the rhizosphere sediments of *P. australis*, *T. angustifolia*, and *C. malaccensis* were not clustered with their adjacent nonvegetated sediments, indicating that the community structures of AOB in the rhizosphere and nonvegetated sediments of *P. australis*, *T. angustifolia*, and *C. malaccensis* were different. In summary, *K. obovata* showed almost no effect on the community structures of all AOPs. *T. angustifolia* had a strong effect on the community structures of all AOPs. *P. australis* and *C. malaccensis* had a certain effect on the community structure of AOB but little effect on the community structures of anammox bacteria and AOA.

### Abundance of anammox bacterial 16S rRNA genes and archaeal and bacterial *amoA* genes

**Fig. 3:**
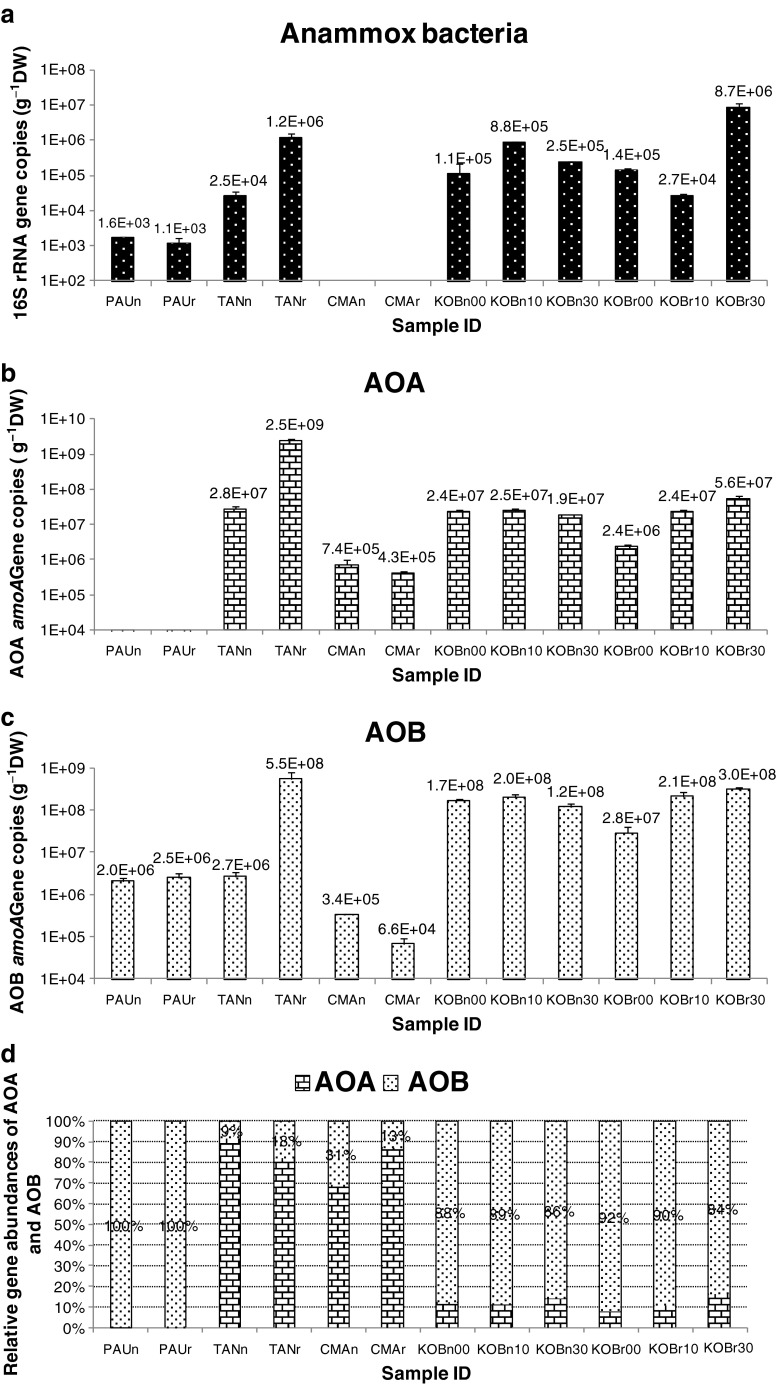
Abundances of 16S rRNA genes and archaeal and bacterial *amoA* genes in the vegetated and nonvegetated sediments of the freshwater and coastal wetlands. For the sample ID, *PAU* indicates the site with *P. australis*, *TAN* indicates the site with *T. angustifolia*, *CMA* indicates the site with *C. malaccensis*, *KOB* indicates the site with *K. obovata*, *r* indicates rhizosphere or vegetated sediment, *n* indicates nonvegetated sediment, and the last two numbers indicate the depth of the sample

Generally, anammox bacterial 16S rRNA gene abundances in the coastal wetland were higher than those in the freshwater wetland (Fig. 3a). Furthermore, the genes were distributed relatively evenly among different samples in the coastal marine wetland than in the freshwater wetland. In the coastal marine wetland, anammox bacterial 16S rRNA gene abundances were between 2.7 × 10^4^ and 8.7 × 10^6^ gene copies g^−1^ DW. In the freshwater wetland, the gene abundances differed significantly at the three sites. No anammox bacteria were detected at the *C. malaccensis* site, but there were up to 1.2 × 10^6^ gene copies g^−1^ DW in the rhizosphere of *T. angustifolia*.

AOA *amoA* genes were distributed relatively evenly between the different samples in the coastal wetland compared to the freshwater wetland (Fig. 3b). The abundances of AOA *amoA* genes in the coastal wetland were between 2.4 × 10^6^ and 5.6 × 10^7^ gene copies g^−1^ DW. AOA *amoA* gene abundances varied significantly between different sites in the freshwater wetland. AOA *amoA* genes were undetectable at the *P. australis* site; however, they reached 2.5 × 10^9^ gene copies g^−1^ DW in the rhizosphere of *T. angustifolia*, almost two orders as high as those of the coastal wetland samples.

The abundances of AOB *amoA* genes were distributed relatively evenly in the coastal marine wetland, between 2.8 × 10^7^ and 3.0 × 10^8^ gene copies g^−1^ DW (Fig. 3c). AOB *amoA* gene abundances varied greatly between the different sites in the freshwater wetland, between 3.4 × 10^5^ and 5.5 × 10^8^ gene copies g^−1^ DW. With the exception of the rhizosphere of *T. angustifolia*, samples of freshwater wetland generally had lower abundances than samples of the coastal wetland.

The relative abundances of AOA and AOB *amoA* genes in each sediment sample in the two wetlands are shown in Fig. 3d. The relative abundances of AOA and AOB *amoA* genes were different between the two wetlands. In the freshwater wetland, with the exception of the *P. australis* site where AOA *amoA* genes were undetectable, the proportion of AOA *amoA* genes was between 69 and 91 %, dominating over AOB *amoA* genes in all the samples of the other two sites. In the coastal marine wetland, however, the proportion of AOB *amoA* genes was between 84 and 92 %, dominating over AOA *amoA* genes in all samples.

Taken together, the abundances of anammox bacterial 16S rRNA genes and AOA and AOB *amoA* genes varied significantly between different sites in the freshwater wetland, but they were relatively evenly distributed in different sediment samples of the coastal wetland. The abundances of the AOPs gene copies in the freshwater wetland, except for the site of *T. angustifolia*, were lower than those of the coastal wetland. AOA *amoA* genes dominated over AOB *amoA* genes in two out of the three sites in the freshwater wetland. While in the coastal wetland, AOB *amoA* genes dominated over AOA *amoA* genes in all sediment samples.

## Discussion

### Physiochemical characteristics of the sediments

The characteristics of sediments in the coastal wetland and the freshwater wetland were quite different (Table 1), indicating that wetland type had an important effect on the characteristics of the sediments. The main reason for the differences is that the two wetlands were respectively under the different effects of seawater and freshwater. Seawater has higher salinity, conductivity, and turbidity than freshwater; therefore, the overlying water of the coastal wetland has higher salinity, conductivity, and turbidity than the freshwater wetland. Seawater is usually slightly alkaline, whereas the freshwater mainly from the runoff of adjacent hills is usually acidic. As a result, sediments in the coastal wetland have higher pH values than the freshwater wetland. The different sampling strategies may also contribute to the differences of sediment characteristics. In the freshwater wetland, the samples were taken from the rhizosphere and nonvegetated sediments at the depth of approximately 10 cm. In the coastal wetland, however, the samples were taken from layers of different depths (0–40 cm in depth) due to homogeneity. In the deeper layers of the nonvegetated sediments in the coastal wetland, NH_4_^+^ concentration was relatively high because no plant roots reached there to absorb them. As a result, the average NH_4_^+^ concentration of all samples in the coastal wetland was apparently higher. Deeper layers usually were more anoxic, so redox potentials of the sediments in the coastal wetland were lower compared to the freshwater wetland. Taken together, the physiochemical characteristics of the sediments in the two wetlands were quite different because of a number of reasons. The differences in sediment characteristics will subsequently affect the community of the AOPs in the sediments and form different AOP community structures in these two wetlands.

The vegetated sediments (including the rhizospheres) and nonvegetated sediments had different physiochemical characteristics (Table 1), indicating that vegetation had an effect on the sediment characteristics. In this study, vegetated sediments generally showed lower NH_4_^+^ concentrations and lower pH, but higher redox potentials. The differences may have resulted from the activity of plants and microorganisms associated with the rhizospheres. Plants in the vegetated sediments absorb NH_4_^+^, which will result in relatively lower NH_4_^+^ concentrations in the vegetated sediments (Ladygina and Hedlund 2010). Enhanced activity of nitrifying microbes might also be a factor for lowering the NH_4_^+^ concentration observed in the vegetated sediments (Glaser et al. 2010). Plants could change the rhizosphere pH by 1–2 units (Nye 1981). For example, plants release protons when they absorb NH_4_^+^, which could result in the reduction of pH (Ding et al. 2011). Lu et al. (2007) found that mangrove roots could release low-molecular-weight organic acids that resulted in the reduction of pH by 0.2–0.5 units in the rhizosphere. In this study, the lower pH observed in the nonvegetated sediments than the corresponding nonvegetated ones might be due to the activity of plant roots and respiration. Plants could also release the excessive oxygen into the rhizosphere (Nikolausz et al. 2008; Soda et al. 2007), which resulted in the higher redox potentials of the vegetated sediments than the corresponding nonvegetated ones in the freshwater wetland. Interestingly, redox potentials in the coastal wetland did not show obvious differences between the vegetated and nonvegetated sediments. Unlike the freshwater wetland, samples taken from the vegetated sites in the coastal wetland were not directly from the rhizospheres but from different layers due to the small mangrove trees. The different layers in the vegetated site were not strongly affected by the direct influence of roots because *K. obovata* in the coastal wetland were still too small (approximately 1.0 m in height) and the plant roots were not dense enough in the sediments. Because of the activity of roots and rhizosphere microorganisms associated, vegetated sediments had a different physiochemical environment from nonvegetated ones, which will in turn affect the community of the AOPs.

### Phylogeny of AOPs in the coastal and freshwater wetlands

In the present study, almost all the clones of anammox bacteria retrieved from the two wetlands were affiliated with the species *Ca.* Scalindua brodae. The results agree with previous observations that two different genera of anammox bacteria were seldom found in the same habitat, suggesting that different anammox bacterial genera had different niche specificity (Humbert et al. 2009). The genus *Ca.* Scalindua was first found in wastewater treatment plant and subsequently detected widely in marine environments (Penton et al. 2006; Schmid et al. 2007). The predominant *Ca.* Scalindua brodae found in the present study was also the major component in Lake Tanganyika (Schubert et al. 2006), indicating that *Ca.* Scalindua could adapt to different environments. In addition to *Ca.* Scalindua, freshwater species *Ca.* Kuenenia stuttgartiensis was also reported to be able to gradually adapt to salty water (Kartal et al. 2006). Besides *Ca.* Scalindua, three clones belonging to the cluster Y were also retrieved in this study. Cluster Y have been reported before, and many uncultured clones affiliated with this cluster have been retrieved from different habitats, including the South China Sea (Hong et al. 2011), aquifer water, hypersaline water, and others (Hirsch et al. 2011). However, no pure culture or enrichment has been acquired in the laboratory and their physiology remains unknown. Some studies suggested that clones in cluster Y are not anammox bacteria and their amplification was due to the primers’ nonspecificity (reviewed by Jetten et al. 2009).

Wetland types showed a pronounced effect on the AOA phylotype composition. All clones of AOA in the coastal wetland fell into clusters 1 and 4 within the sediment clade. This is in agreement with early classification because of the marine nature of the coastal wetland. However, the clones of AOA from the freshwater wetland fell into different clades. As shown in previous studies, AOA in the terrestrial ecosystem usually belong to soil/sediment clade (Zhang et al. 2008). The freshwater wetland in this study, however, in addition to the soil/sediment clade, also contained species of sediment clade, and even species of water column clade. This finding challenges previous knowledge and suggests that species in the marine system could also survive in the freshwater wetland. From this point of view, AOA might adapt to a much wider range of habitats. With further studies on AOA from different habitats, more and more species previously believed to only exist in one type of habitat might also occur in other types of habitats (Pester et al. 2012). Although the established scheme by Francis et al. (2005) could correlate AOA species with their ecophysiological characteristics, this method might not be suitable for new findings in the future. A new classification method for AOA has been proposed recently, in which AOA were classified into five phylogenetic clusters named after the first genus found in that cluster (Pester et al. 2012).

The dominant AOB phylotypes in the two wetlands were affiliated with the *Nitrosospira*-like genus. In the *Nitrosospira*-like genus, all the retrieved sequences were affiliated with two unknown clusters: clusters 1 and 2. Although uncultured clones have been reported before in clusters 1 and 2, no pure cultures of them have been acquired to date. The two clusters might be two new uncultured species, which are widely distributed in natural environments. Previous studies have shown that *Nitrosospira* and *Nitrosomonas* favor different habitats. *Nitrosospira* favor clean water of low ammonia, while *Nitrosomonas* favor polluted environments of high ammonia and frequently in wastewater treatment plants (Okabe et al. 1999). In the present study, the two wetlands were dominated by *Nitrosospira*-like, suggesting that the two wetlands were not strongly affected by wastewater. Although most *Nitrosomonas* favor environments of high NH_3_, *Nitrosomonas marina* usually exist in marine environments of low NH_3_ (Pommerening-Röser et al. 1996). In this study, most of the *Nitrosomonas*-like sequences retrieved from the two wetlands were close to *N. marina*, which further suggests that the two wetlands were not affected by intense anthropogenic pollution. Although *Nitrosospira*-like dominated both wetlands, the community compositions of AOB in the two wetlands were quite different (Fig. 1c). The major dominant AOB in the coastal wetland was *Nitrosospira*-like. However, in the freshwater wetland, although the majority belonged to *Nitrosospira*-like, more than one third belonged to *Nitrosomonas*-like.

### Diversity and community structures of anammox bacteria, AOA, and AOB

As discussed above, the freshwater wetland had broader AOP phylotypes than the coastal wetland. In addition to that, the diversities of AOPs in the freshwater wetland were also higher than those in the coastal wetland (Table 2). Furthermore, community structures of AOPs in the two wetlands were also different, as shown in PCoA results (Fig. 2). All these suggested that the wetland type had a strong influence on AOP community structures. Sediments of the coastal and freshwater wetlands had distinct physiochemical characteristics (Table 1), which might be responsible for the differences in the community structures of AOPs. The coastal wetland was restored from deserted salt pans approximately 40 years ago and planted with *K. obovata*. Because the restoring time was relatively short, *K. obovata* were still small (about 1 m in height). Therefore, the effects of *K. obovata* on AOPs were weak, but were evident at Mai Po Nature Reserve in Hong Kong where mangrove trees were more than 100 years old (Li et al. 2011b). Furthermore, this wetland and the associated microorganisms were under regular tidal activity. Because of the relative homogeneity of the ecosystem and regular environmental factors, AOP communities were relatively simple. In the freshwater wetland, however, the situation was different. This wetland was transformed from fishponds. The input water was surface runoff from adjacent hills and wastewater from nearby villages. Different parts of the wetland were planted with different aquatic plants. All these made the ecosystem of the freshwater wetland more complex and heterogeneous, and the environmental factors were also very variable. As a result, the anammox bacterial community of the freshwater wetland was more complex than the coastal wetland.

### Effects of different plants on the phylogeny and community structures of AOPs

Studies of mangrove sediments (Li et al. 2011b) and paddy soils (Wang and Gu 2012) indicated vegetation of plants might have an effect on anammox bacteria. For AOA, some studies showed plants affected the community composition (Chen et al. 2008; Herrmann et al. 2008), but others showed no effects at all (Herrmann et al. 2009). For AOB, some studies showed effects on the community composition (Briones et al. 2002; Chen et al. 2008; Herrmann et al. 2008), but others did not (Herrmann et al. 2009; Kowalchuk et al. 1998; Nicolaisen et al. 2004). In general, the effects of plant roots on the community composition in the rhizospheres are still controversial. In the freshwater wetland of the present study, *T. angustifolia* affected the community structure of anammox bacteria, but *P. australis* did not (Fig. 2a, b). For AOA, *T. angustifolia* had a strong effect and *C. malaccensis* had a weak one on the community structure (Fig. 2c, d). *P. australis*, *T. angustifolia*, and *C. malaccensis* all showed an influence on the community structure of AOB (Fig. 2e, f).

As plants could release exudates such as organic acids and transport oxygen into the rhizospheres (Lu et al. 2007; Nikolausz et al. 2008), the physiochemical characteristics of the rhizospheres are usually different from those of the nonvegetated sediments. In addition to the redox potentials, other factors such as NH_4_^+^ and NO_2_^−^ concentrations in the rhizospheres were also different from the nonvegetated regions. Different plants have different abilities to change the characteristics of rhizospheres and, therefore, the community composition of AOPs. Anammox bacteria occur in oxygen-limited environments, such as oxic/anoxic interfaces (Jetten et al. 2009). Between the rhizospheres and bulk sediments, an oxic/anoxic interface exists. As a result, anammox bacterial community in the rhizospheres was different from that of nonvegetated areas (Li et al. 2011b). The oxic rhizosphere benefits AOA and AOB because these microorganisms frequently occur under oxic environments. *T. angustifolia* is exotic to Hong Kong. It is a fast-growing plant which has strong metabolism and changes the characteristics of the rhizosphere. In this study, *T. angustifolia* had a strong effect on the community structures of all AOPs in the rhizospheres. In addition to *T. angustifolia*, *P. australis* and *C. malaccensis* also showed a certain degree of effects on the community structures of AOB (Fig. 2).

Despite the fact that, in the freshwater wetland, some plants showed effects on community structures of AOPs, in the coastal wetland, *K. obovata* had no apparent effect on the community structures of all AOPs (Fig. 2). *K. obovata* trees growing in the coastal wetland were still small and the sediment samples in the vegetated site of *K. obovata* were from different layers rather than directly from the rhizospheres. As a consequence, the effect of *K. obovata* on AOP community might be weak in this study. Because of that, we could not exclude the possibility that larger *K. obovata* trees might have obvious effects on AOP community structures in the rhizospheres.

### Abundance of anammox bacterial 16S rRNA genes and archaeal and bacterial *amoA* genes

Abundances of AOPs in the coastal wetland were relatively evenly distributed between the sediment samples, whereas abundances of AOPs in the freshwater wetland were unevenly distributed by sites (Fig. 3). This is because the sediment samples in the coastal wetland were relatively homogeneous, while those in the freshwater wetland were very heterogeneous. In the coastal wetland, all sites were under the same effect of tidal activity and the sediments were quite homogeneous. Although vegetation had an effect on the sediments, the mangrove trees were still too small and the effect was not significant enough. In the freshwater wetland, water was collected and treated prior to being discharged into the wetland and the characteristics of the water were gradually changing from the inlet to the outlet of the wetland due to the self-purification. Abundances of AOPs varied from the inlet to the outlet accordingly. Specifically, AOA were undetectable in the *P. australis* site near the inlet of the wetland, and anammox bacteria were undetectable in the *C. malaccensis* site at the outlet. Ammonium concentration was relatively higher in the upstream than the downstream because of the treatment in action. Previous studies have shown that anammox bacteria favor high ammonium and AOA favor low ammonia. That is probably the reason AOA were undetectable in the upstream and anammox bacteria were undetectable in the downstream in the freshwater wetland.

Anammox bacterial 16S rRNA gene copies were relatively higher in the coastal wetland than in the freshwater wetland. Previous studies showed that anammox bacteria only exist in certain habitats, suggesting that some conditions such as oxic/anoxic interfaces and ammonia concentration are important factors determining the existence of anammox bacteria in natural environments (Jetten et al. 2009). Because of the regular influence of tidal activity, the sediments of the coastal wetland had more chances to form oxic/anoxic habitats. Furthermore, the average NH_4_^+^ concentration in the coastal wetland samples was higher than that in the freshwater wetland. As a consequence, the coastal marine wetland was more suitable for anammox bacteria to survive. Generally, freshwater wetland had lower AOB abundances than the coastal wetland, probably because sediment samples of the former had a lower ammonia concentration than the latter. AOA abundances in the freshwater wetland varied remarkably in different sites. In the *C. malaccensis* site, no AOA were detected. In the *T. angustifolia* site, AOA abundance was even higher than those of the coastal marine wetland. This result suggests that many factors codetermine the abundances of AOA.

Since the discovery of AOA, the relative importance of AOB or AOA in the ecosystem became a heated debate. AOB were found to numerically dominate over AOA in some habitats (Di et al. 2009), whereas AOA were found to outnumber AOB in many other environments (Adair and Schwartz 2008; Boyle-Yarwood et al. 2008; Cao et al. 2011b; Radax et al. 2012; Schauss et al. 2009). Although a significant number of studies emphasized the numerical competence of AOA, only four studies linked the nitrification rate with AOA abundance (reviewed by Pester et al. 2011). Furthermore, numerical dominance does not certainly represent functional importance, for example, Jia and Conrad (2009) demonstrated that, although AOA *amoA* genes numerically dominated an agricultural soil, AOB were responsible for ammonia oxidation. In the present study, AOA dominated over AOB in the freshwater wetland, with the exception of the *P. australis* site at the inlet where NH_4_^+^ concentration of water was relative high. In the coastal wetland, however, AOB dominated over AOA. The dominance of AOB or AOA in the environments is believed to be determined by some factors, such as ammonia concentration, pH, and organic matter. AOA have been demonstrated to be more competitive in low NH_3_ concentration because of their high affinity (Martens-Habbena et al. 2009). Some AOA species, such as *Ca.* Nitrosotalea devanaterra, were demonstrated to favor low pH environments (Lehtovirta-Morley et al. 2011; Yao et al. 2011). Although both AOB and AOA were demonstrated to be chemolithoautotrophic organisms, some studies suggested that AOA are mixotrophic or even heterotrophic (Jia and Conrad 2009). In the present study, NH_4_^+^ concentration and pH in the freshwater wetland were relatively lower than the coastal marine wetland, which are probably the reasons why AOA dominated the freshwater wetland and AOB dominated the coastal wetland. Although organic matter was not measured, the freshwater wetland in this study might have higher organic matter than the coastal wetland, which also facilitated the AOA existence in the freshwater wetland.

Due to the distinct physiochemical properties of the rhizospheres, communities of AOPs in the rhizospheres tend to be different from those in the bulk soils or nonvegetated soils. Herrmann et al. (2008) discovered that, in the rhizosphere of *Littorella uniflora*, AOA *amoA* genes were much more enriched compared with AOB *amoA* genes, suggesting that AOA were responsible for the enhanced nitrification activity observed in the rhizosphere. Subsequently, they further confirmed that *L. uniflora* and *Juncus bulbosus* harbored higher AOA abundances in the rhizospheres than in the nonvegetated soils (Herrmann et al. 2009). Another study showed that vegetation of *Spartina alterniflora* increased AOB numbers but reduced AOA numbers (Zhang et al. 2011). Studies on the effects of the rhizospheres on anammox bacteria are relatively scanty; Li et al. (2011b) suggested that mangrove trees might influence anammox bacterial abundances in the sediments. In the present study, *K. obovata*, *P. australis*, and *C. malaccensis* seemed to have no detectable effects on the abundances of anammox bacteria, AOA, or AOB since the vegetated and nonvegetated sediments showed similar abundances of AOPs (Fig. 3). However, *T. angustifolia* seemed to be a special species. The abundances of anammox bacteria, AOA, and AOB in the rhizosphere of *T. angustifolia* were relatively higher than the nonvegetated sediment. This result suggested that *T. angustifolia* could enhance AOPs’ quantity in the rhizosphere. In the future, it might be important to study why the exotic *T. angustifolia* enhances the quantity of AOPs.

In conclusion, AOPs in the vegetated and nonvegetated sediments in a coastal marine wetland and a freshwater wetland were investigated. The more heterogeneous freshwater wetland had a broader range of phylotypes, higher diversity, and more complex community structures of AOPs than the coastal marine wetland. Generally, the effects of vegetation on the community structures of AOPs were macrophyte species-specific. Vegetation of *T. angustifolia* had an apparent effect on the community structures of all AOPs. Vegetation of *P. australis* and *C. malaccensis* had some effect on the community structure of AOB but had little effect on the community structures of anammox bacteria and AOA. *K. obovata* showed almost no effect on the community structures of all AOPs. Information from this study allows further assessment of nutrient removal by wetland and the related functional groups of microorganisms responsible for the treatment efficiency. In addition, better management of wetland may be achieved through fundamental understanding of the microbial community structures.

## Electronic supplementary material

Below is the link to the electronic supplementary material.

ESM 1 (PDF 400 kb)
