# Supplementary material for: Higher diversity of ammonia/ammonium-oxidizing prokaryotes in constructed freshwater wetland than natural coastal marine wetland
Source: Appl Microbiol Biotechnol. 2012 Oct 9;97(15):7015–33. doi: 10.1007/s00253-012-4430-4 (PMC3708290; doi:10.1007/s00253-012-4430-4)
Supplement: Supplementary file 1 — (PDF 400 kb) [file 253_2012_4430_MOESM1_ESM.pdf]

Supplementary materials:

Higher Diversity of Ammonia-Oxidizing Prokaryotes (AOPs) in Constructed Freshwater Wetland than Natural Coastal Wetland

Yong-Feng Wang[1] and Ji-Dong Gu[1,2]*

[1] Laboratory of Environmental Microbiology and Toxicology, School of Biological Sciences, The University of Hong Kong, Pokfulam Road, Hong Kong SAR, People's Republic of China

[2] The Swire Institute of Marine Science, The University of Hong Kong, Shek O, Cape d'Aguilar, Hong Kong SAR, People's Republic of China

* Corresponding author: Tel.: (+852) 2299-0605; fax: (+852) 2559-9114

E-mail address: jdgu@hkucc.hku.hk

Figure S1 Locations of the coastal wetland and the constructed freshwater wetland in Hong Kong. The coastal wetland and the freshwater wetland are located in Tai O and Yuen Long, respectively. Both of them are indicated with stars in red color on the map.

.

Figure S1

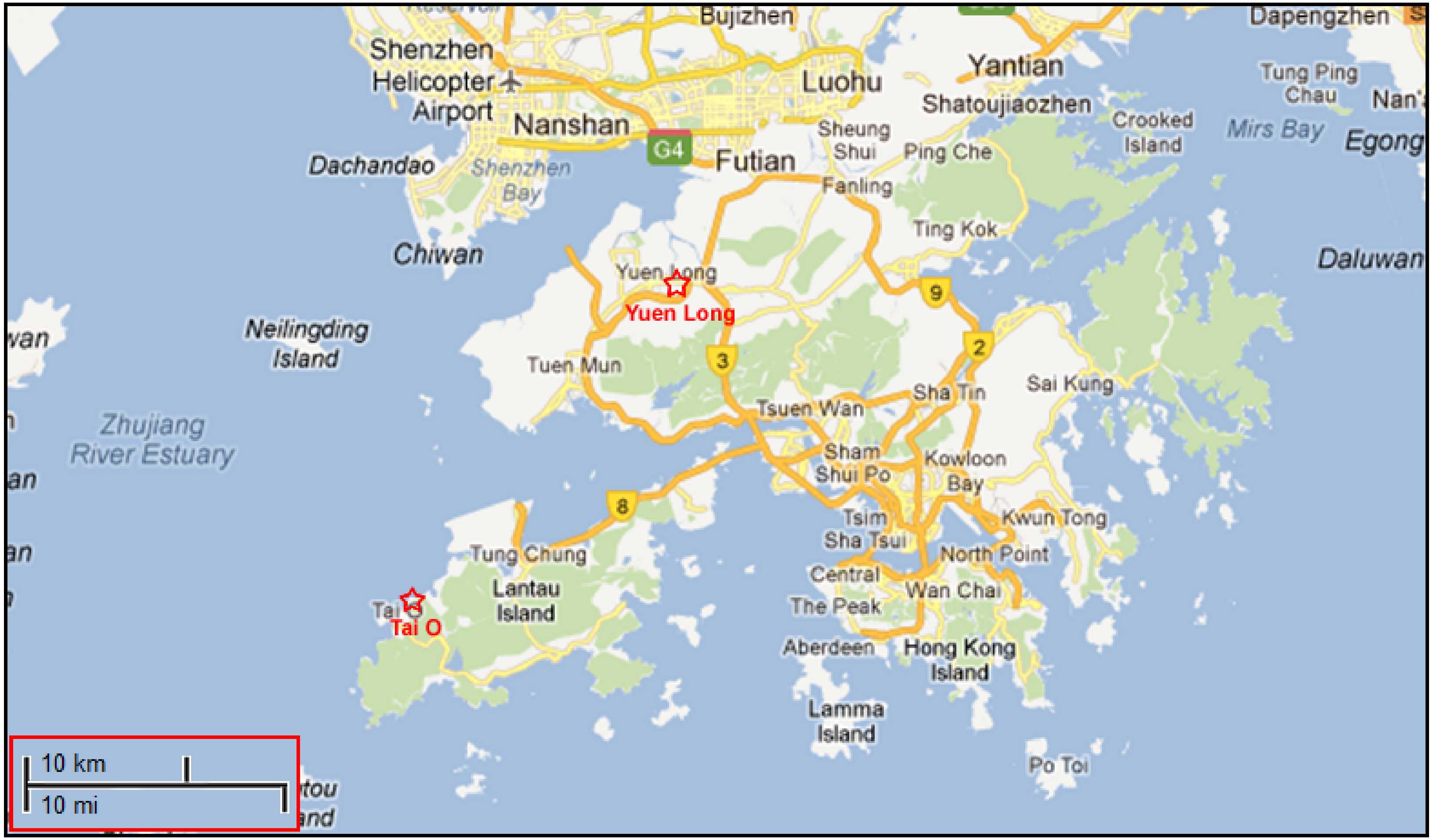

Bujizhen
Dapengzhen
Shenzhen
Helicopter
Airport
Nanshan
Luohu
Yantian
Shatoujiaozhen
Tung Ping
Chau
Crooked
Island
Mirs Bay
Egong
G4
Futian
Sheung
Shui
Ping Che
Dachandao
Shenzhen
Bay
Fanling
Ting Kok
Chiwan
Daluwan
Yuen Long
Yuen Long
9
2
Neilingding
Island
Tuen Mun
3
Sai Kung
Sha Tin
Tsuen Wan
Zhujiang
River Estuary
Sham
Shui Po
Kowloon
Bay
8
Tsim
Sha Tsui
Kwun Tong
Tung Chung
North Point
Central
Lantau
Island
Wan Chai
Tai O
Tai O
The Peak
Aberdeen
Hong Kong
Island
Lamma
Island
Po Toi
10 km
10 mi

Figure S2 Rarefaction curves indicating 16S rDNA richness of (a) anammox bacteria, (b) archaeal *AmoA* richness and (c) bacterial *AmoA* richness. OTUs were defined at 1% differences in nucleotide sequence for anammox bacteria and 3% difference in deduced amino acid sequences for AOA and AOB. PAU is for the site grown with *Phragmites australis*, TAN with *Typha angustifolia*, CMA with *Cyperus malaccensis*, ‘r’ indicates the rhizosphere, ‘n’ indicates the non-vegetated sediments. The number indicates the sampling depth.

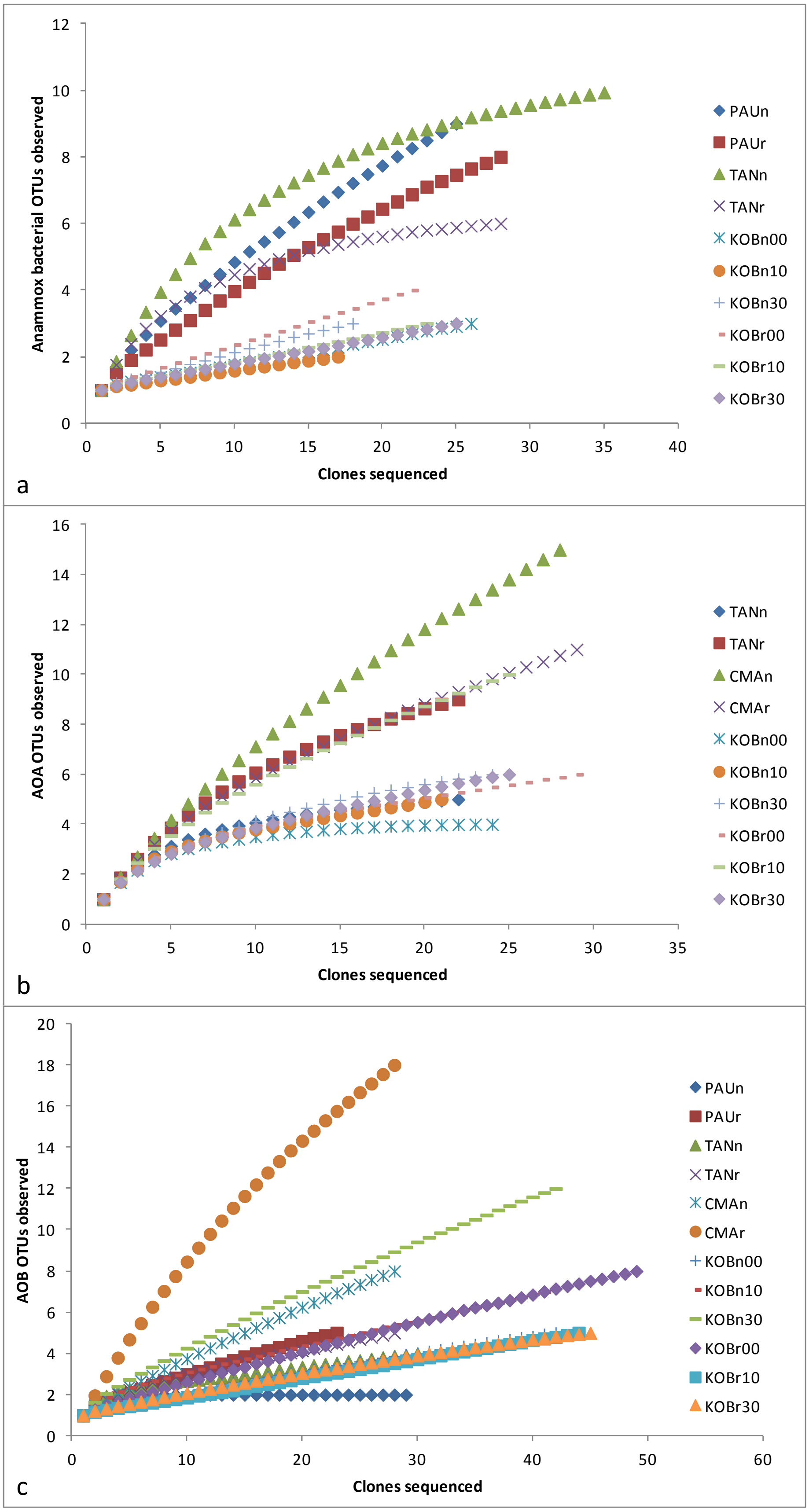
Anammox bacterial OTUs observed
Clones sequenced
PAUn
PAUr
TANn
TANr
KOBn00
KOBn10
KOBn30
KOBr00
KOBr10
KOBr30
a
AOA OTUs observed
Clones sequenced
TANn
TANr
CMAn
CMAr
KOBn00
KOBn10
KOBn30
KOBr00
KOBr10
KOBr30
b
AOB OTUs observed
Clones sequenced
PAUn
PAUr
TANn
TANr
CMAn
CMAr
KOBn00
KOBn10
KOBn30
KOBr00
KOBr10
KOBr30
c
